# Differential Effect of Growth Factors on Invasion and Proliferation of Endocrine Resistant Breast Cancer Cells

**DOI:** 10.1371/journal.pone.0041847

**Published:** 2012-07-30

**Authors:** Maitham A. Khajah, Sanaa Al Saleh, Princy M. Mathew, Yunus A. Luqmani

**Affiliations:** 1 Faculty of Pharmacy, Kuwait University, Safat, Kuwait; 2 Faculty of Medicine, Kuwait University, Safat, Kuwait; II Università di Napoli, Italy

## Abstract

We have established several breast cancer cell lines that exhibit a permanent ER-depleted phenotype, induced by shRNA transfection of MCF-7 cells, which afford a useful model for studying acquired endocrine resistance. Previously we showed that MDA-231 as well as ER-silenced cells could invade through simulated extracellular matrix components. However, the contribution of individual serum components responsible for cell invasion was not determined. In the present study, an under-agarose gel assay was used to quantitatively assess the invasive movement of two ER-silenced cell lines (pII and YS2.5) in comparison to the parental MCF-7, the ER negative MDA-231, and normal HBL100 cells, as well as a line that was ER-shRNA transfected but failed to exhibit ER down-regulation (YS1.2). We also examined the effect of the growth factors EGF, IGF-1, TGFβ, PDGFC and RANTES on pII cell invasion and proliferation. All breast cancer cell lines which had reduced ER expression exhibited a serum-dependent invasive ability related to the degree of induced ER loss. TGFβ treatment inhibited pII cell proliferation and enhanced their invasive ability but at a relatively high dose. IGF-1 and EGF enhanced pII cell proliferation, with the latter playing the major role in promoting cell invasion. PDGFC did not affect either process although it is highly expressed in pII cells. Differential effects were observed on activation of Akt and ERK1/2 suggesting their involvement as intracellular mediators of EGF induced invasion, in part through the regulation of matrix metalloproteinase activity. Targeting EGF receptor tyrosine kinase activity by erlotinib resulted in significant inhibition of both pII cell proliferation and directional invasion towards EGF suggesting that this drug has potential therapeutic usefulness for preventing spread of particularly endocrine resistant breast cancer.

## Introduction

Tamoxifen has formed the basis of treatment of women with metastatic breast cancer [1] for several decades, resulting in significant improvements in quality of life and overall survival rates [2] in a significant proportion of patients with clinically defined positive estrogen receptor (ER) status. However, both *de novo* and *acquired* resistance to tamoxifen, as well as to other endocrine agents, due to the loss of tumoural ER expression and/or its function presents a major therapeutic challenge, and usually leads to more aggressive disease upon relapse [3]. Cellular transition from an epithelial to a mesenchymal phenotype (epithelial to mesenchymal transition; EMT) has been identified in various disease conditions including breast neoplasia [4] and is associated with endocrine resistance and poor prognosis [4]–[6]. Accompanying phenotypic changes include loss of cell-cell adhesion as a result of reduced E-cadherin and expression of catenins within adherens junctions, reduced claudins and occludins expression at tight junctions and reduced expression of epithelial cytokeratins such as KRT8, 18, and 19 which presumably aids in disruption of cytoskeletal connections necessary for maintaining normal tissue architecture. A variety of growth factors and their downstream signaling components have been associated with endocrine resistance and EMT. These include transforming growth factor β (TGFβ), insulin like growth factor 1 receptor (IGF1R), epidermal growth factor receptor (EGFR), PI3K/Akt, ERK/MEK, and MAPK [3], [6]–[13].

We have previously reported the establishment of several cell lines in long term culture that have switched from an estrogen responsive to an endocrine independent state by the depletion of ER, induced by shRNA transfection of MCF-7 cells [14], [15]. Microarray and real time-PCR analysis confirmed a modified gene expression profile in the established transfectant cells indicative of EMT; loss of epithelial markers including E-cadherin, catenin, occludins and claudins, and enhanced expression of genes normally associated with mesenchymal cells such as N-cadherin, vimentin, fibronectin, integrin β4 and α5, and various metalloproteinase. In addition, these ER-depleted cells exhibit a series of changes in morphology and enhanced motility and invasiveness [14]. It was demonstrated that MDA-231 cells (derived from a *de novo* ER negative breast tumor) as well as shRNA mediated ER-depleted cells (pII) (but not the parental ER positive MCF-7) were able to invade through a layer of basement membrane protein extract that is widely used to simulate the extracellular matrix (ECM). We also used agarose spots to simulate the polysaccharide component of the ECM and showed that pII cells could also penetrate into such structures. These experiments were conducted in the presence of serum; the contribution of individual serum components responsible for cell invasion was not determined.

In the present study, we used an under-agarose gel assay [16]–[18] to study the invasive movement of two of the ER- depleted cell lines (pII and another designated YS2.5 which displays enhanced ER silencing compared to pII) in comparison to the parental MCF-7 cells, MDA-231, the normal breast epithelial cell line HBL100, as well as a line that was ER-shRNA transfected but failed to exhibit ER down-regulation (YS1.2). We present data on the effect of the growth factors EGF, IGF-1, TGFβ, PDGFC and the chemokine RANTES on cell invasion and proliferation. We demonstrate that EGF is the most effective stimulus for endocrine resistant cells to invade into an agarose matrix, that this is associated with increased phosphorylation of Akt and ERK1/2 and at least in part mediated by increased metalloproteinase (MMP) activity.

## Materials and Methods

### Cell Lines

HBL100 normal breast epithelial cell line, MCF-7 and MDA-231 human breast carcinoma cell lines were obtained from the ATCC (American Type Culture Collection, VA, USA). pII, YS2.5 and YS1.2 cell lines were established in this laboratory by transfection of MCF-7 with ER directed shRNA plasmid as described previously [14], [15]. For routine culture all cell lines were maintained at 37°C in a humidified atmosphere of 5% CO_2_ in advanced DMEM supplemented with 5% fetal bovine serum (FBS), 600 µg/ml L-glutamine, 100 U/ml penicillin, 100 µg/ml streptomycin and 6 ml/500 ml 100 x non-essential amino acids (all from Invitrogen, CA, USA). For YS2.5 and YS1.2, the maintenance medium also contained G418 (1 mg/ml) but this was omitted during experiments.

IGF-1, EGF, TGFβ and the CC chemokine RANTES were purchased from Sigma, USA. PDGFC was purchased from Sino Biologicals. Stock solutions were prepared in sterile PBS and stored in small aliquots at −20°C and diluted in sterile PBS just prior to performing experiments. The EGF inhibitor erlotinib (LC laboratories, USA), the ERK inhibitor PD0325901 (Chemitek), and the Akt inhibitor LY294002 (Tocris Bioscience) were prepared in sterile dH_2_O and stored in small aliquots at −20°C and diluted in DMEM just prior to performing experiments.

### Cell Invasion Assay

**Figure 1:**
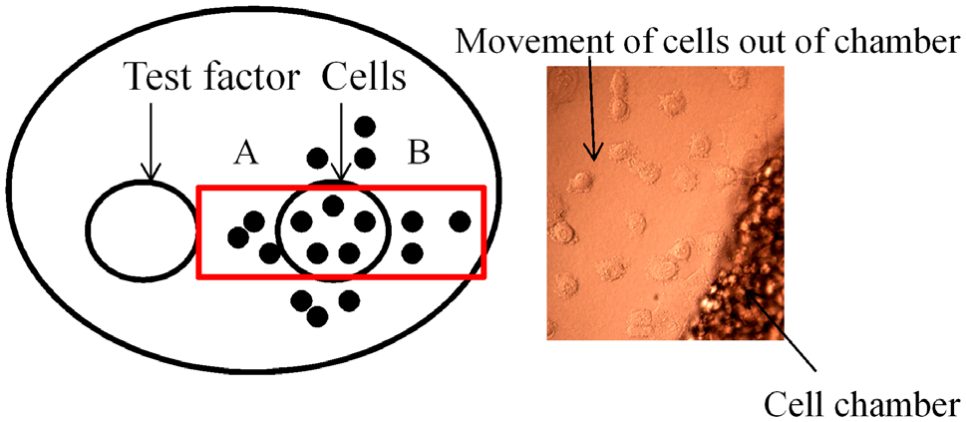
The under-agarose assay. The schematic diagram (left) demonstrates the general plate setup in all the cell invasion assay experiments described in subsequent figures. Two wells (sometimes three) were created in the agarose layer formed in a 6 well dish as described in Methods; one containing cells and the other the chemoattractant or test factor. Only cells moving into the area indicated by the red box were counted for both random (A + B) and directional net (A – B) invasion through the agarose layer. The micrograph on the right (x10 magnification) illustrates actual cells that have penetrated out of the cell chamber into the surrounding agarose. In all experiments, cells inside the chamber were suspended in complete medium containing 5% serum, without which invasion was not observed.

Ultra-pure agarose (Invitrogen) was melted in PBS, then (once cooled below 40°C) supplemented with DMEM with or without 5% FBS or other components (insulin-transferrin-selenium (ITS) (Invitrogen), ITS plus bovine serum albumin (BSA) (Sigma) or IGF-1 to give a final 0.5% solution and allowed to solidify in individual wells of 6 well dishes at room temperature. Once set, 1–3 sample chambers (3.5 mm in diameter) were created in the gel, 2.5 mm apart in a horizontal line, by insertion of a metallic mould. Cells (4×10^4^) were re-suspended in DMEM containing various additives (5% FBS, 600 µg/ml L-glutamine, 100 U/ml penicillin, 100 µg/ml streptomycin and 6 ml/500 ml 100 x non-essential amino acids) and loaded into formed chambers as appropriate for the experiment. Plates were incubated at 37°C in 5% CO_2_ humidified atmosphere. After 24 h, cells that had penetrated into the agarose were manually counted by visual microscopic examination. Random cell invasion was determined as the total number of cells which moved in both lateral directions (A+B). Directional cell invasion was determined by subtracting B from A (Fig. 1). All measurements were performed at least in triplicate and each experiment performed at least twice on separate days. It should be noted that cells did move in all directions around the chamber but only the lateral movement was considered in order to standardize the quantitation.

Experiments were set up in the following manner: (1) a single cell chamber was created in the agarose and the cell line tested was added to the chamber (4×10^4^ cells in 10 µl media) and lateral random invasion (A + B) through the agarose layer which contained 5% FBS or other serum components was determined. (2) two chambers (3.5 mm in diameter) were created 2.5 mm apart and the lateral directional (A – B) invasion of the cells from one chamber towards the source of the test factor present in the other chamber was determined. (3) one chamber was created and the lateral random movement (A+B) of cells out of the chamber was determined by adding the test factor directly to the cells. (4) three cell chambers were created (3.5 mm in diameter), 2.5 mm apart; the two outer chambers contained untreated (UT) cells and cells pre-treated with erlotinib, PD0325901 or LY294002 respectively. The directional net cell invasion of treated cells toward EGF (50 ng/ml) present in the middle chamber was determined by subtracting the number of cells that moved out of the chamber with untreated cells. (5) three chambers were created (3.5 mm in diameter, 2.5 mm apart) and the lateral movement of cells placed into the middle chamber toward two growth factors present in the two outer chambers (competition assay) was determined. A schematic diagram is shown with the appropriate figure in the Results section to illustrate the design for each set of experiments.

### Cell Proliferation Assay

The effect of growth factors (EGF, IGF-1, PDGFC and TGFβ) and EGF inhibitor (erlotinib) on pII cell proliferation was examined. Approximately 10^4^ cells were seeded into triplicate wells of 12-well plates and allowed to attach overnight. Either vehicle only or erlotinib (1–10 µM) was then added to the cells. Growth was assessed by MTT assay after 4 days of incubation. Briefly, 1 ml of MTT [3-(4,5-dimethylthiazolyl-2)-2,5-diphenyltetrazolium bromide] reagent (Promega) (0.5 mg/ml) was added to each well and plates incubated at 37°C for 30 min followed by the addition of 1 ml acidic isopropanol and vigorous re-suspension of the converted blue dye. Absorbance of the suspension was measured at 595 nm with background subtraction at 650 nm. The effect of the inhibitor used was compared to untreated control cells (taken as 100%).

To test the effect of growth factors on proliferation, cells were plated as above and after 24 h the medium was replaced with serum-free standard DMEM (in case of using IGF-1, PDGFC or EGF) or 5% FBS containing DMEM (in case of using TGFβ) and left for another 24 h prior to the addition of IGF-1 (2–100 ng/ml), EGF (10–200 ng/ml), PDGFC (1–100 ng/ml), TGFβ (1–10 ng/ml) or vehicle. Cell growth was then determined after a further 4 days as described above.

### Western Blotting

The level of total and phosphorylated Akt and ERK1/2 protein was determined in pII cells in response to IGF-1, EGF and TGFβ (1–100 ng/ml; 5–120 min) stimulation by immunoblotting. pII cells were cultured in 6 well plates with DMEM containing various additives (5% FBS, 600 µg/ml L-glutamine, 100 U/ml penicillin, 100 µg/ml streptomycin and 6 ml/500 ml 100 x non-essential amino acids) until reaching 80% confluence and then serum-starved overnight before addition of growth factors (with vehicle only as control) for periods up to 120 min. Cells were then harvested by scraping, following the addition of 300 µl of lysis buffer containing 50 mM HEPES, 50 mM NaCl, 5 mM EDTA 1% Triton X, 100 µg/ml PMSF, 10 µg/ml aprotinin, and 10 µg/ml leupeptin. Protein assay was determined by the standard Bradford assay and 8 µg protein was mixed with an equal volume of 2 x SDS and heated at 90°C for 10 min. Lysates were loaded onto a 10% SDS-polyacrylamide gel and electrophoresed at 150 V for 1 h. Proteins were transferred to a nitrocellulose membrane and blocked with 2% BSA for 1 h before being incubated overnight at 4°C with either total or pAkt antibody (Ser473 from Cell Signalling, USA) (1/600 dilution), total or pERK1/2 antibody (Abcam, UK) (1/1000 dilution), or actin antibody (Cell Signalling,) (1/1000 dilution) prepared in 2% BSA. The membrane was washed and incubated with anti-HRP-conjugated secondary antibody (Cell Signaling) (1/500 dilution) for 1 h, developed with Super Signal ECL and visualized with Kodak X-ray film.

### Matrix Metalloproteinase (MMP) Activity Assay

The general activity of MMP enzyme was determined using an assay kit purchased from Abcam (Cat No. ab112146) according to the manufacturer’s protocol. In brief, pII or YS1.2 (10^4^ cells) were seeded into triplicate wells of 6-well plates and allowed to attach overnight, then starved with serum free media for another 18 h. Cells were then treated with vehicle (control), IGF-1 or EGF (10–50 ng/ml) for 30 min and the MMP activity was assayed in the conditioned media; for this, 25 µl of medium was removed and added to 25 µl of 2 mM APMA working solution and incubated for 15 min at 20°C followed by addition of 50 µl of the green substrate solution supplied in the kit - a broad spectrum MMP fluorogenic peptide substrate. A kinetic measurement was then performed for the MMP activity by taking medium samples at 5 min intervals over a 1 h period after starting the reaction by using a microplate reader with a filter set of Ex/Em = 490/525 nm. In another set of experiment, pII cells were either un-treated, or pre-treated with PD0325901 or LY294002 (10 µM) for 1 h and then stimulated with EGF (50 ng/ml) for 30 min and MMP activity was measured as described above.

### Statistical Analysis

Student’s two tailed unpaired t- test was used to compare means of individual groups: p≤0.05 was considered statistically significant.

## Results

### Comparative Invasive Movement of Cell Lines Used in this Study

**Figure 2:**
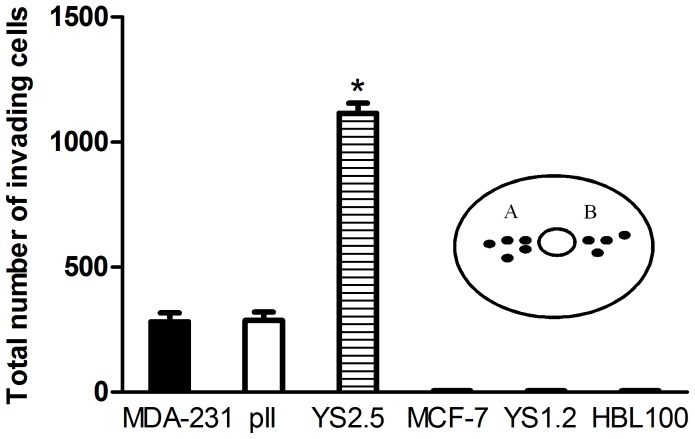
Comparative invasive ability of cell lines used in this study. The total random invasion (A + B) of the cell lines indicated, through the agarose containing 5% serum, was determined as described in Methods and depicted in Fig 1. Histobars are means ± SEM of 11–28 independent determinations. * denotes significant difference from MDA-231 and pII with p<0.0001.

As illustrated in Fig. 2, neither the parental MCF-7 nor the transfected (but not ER-down-regulated) YS1.2 cells were able to penetrate into the agarose. In addition, the normal breast epithelial cell line HBL100 did not show any invasive ability. MDA-231 and pII cells did enter the agarose in all directions and to a similar extent. The greatest penetration was however observed with the YS2.5 cells.

### Effect of Serum Components on Invasive Capacity of MDA-231 and pII Cells

**Figure 3:**
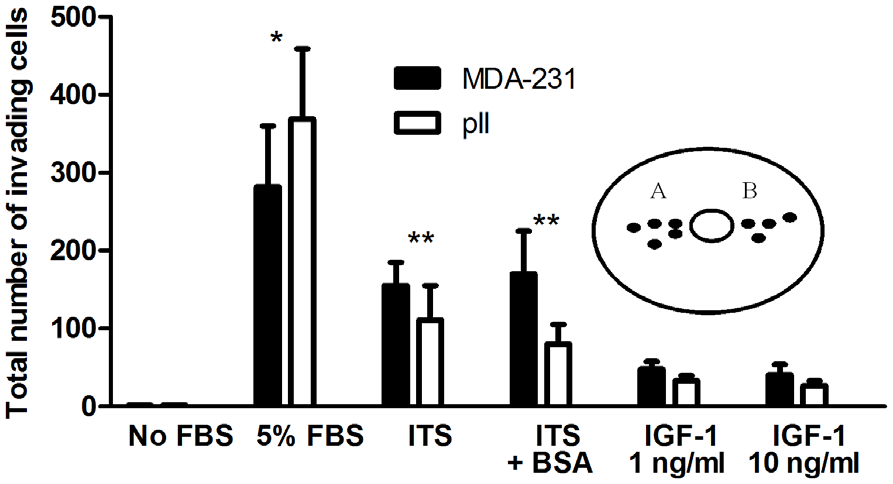
Effect of serum components on invasive capacity of MDA-231 and pII cells. The total invasive movement (A + B; see Fig 1) of MDA-231 and pII cells cultured in DMEM containing serum was determined either in the absence of serum in the agarose, or with the addition of either 5% FBS, insulin-transferrin-selenium-A solution (ITS), ITS + BSA (1.5 mg/ml) or IGF-1. Histobars are means ± SEM for 3–19 independent determinations. For both cell lines, invasion in the presence of serum in the agarose was significantly greater than with all other conditions, with p≤0.05 (*), and ITS with or without BSA had a significantly greater effect than IGF-1, with p≤0.05 (**).

Both MDA-231 and pII cells showed absolute dependence on the presence of serum in the agarose for invasion (Fig. 3). To investigate what minimum components in the serum may be responsible/necessary for the cell movement, the serum was substituted with an ITS solution, which is a commonly employed supplementation to many conventional synthetic nutrient media that permits substantial reduction in the serum requirement for routine maintenance of cells in culture. In the presence of ITS in the agarose, MDA-231 and pII cells both showed some degree of invasion but this was significantly less than with the optimal 5% serum condition. The addition of BSA (1.5 mg/ml) with the ITS did not further increase the degree of invasion of either cell line. To determine if the ITS effect was due to the insulin, the experiment was performed with the addition of IGF-1 to the basic serum-free medium. At 1 and 10 ng/ml IGF-1 elicited some invasive activity but significantly less than that observed with ITS. It should be noted that in all experiments, the cells inside the chamber were suspended in complete medium containing 5% serum, without which invasion was not observed.

### Effect of IGF-1, EGF, TGFβ, PDGFC, and RANTES on the Directional Invasion of pII Cells

**Figure 4:**
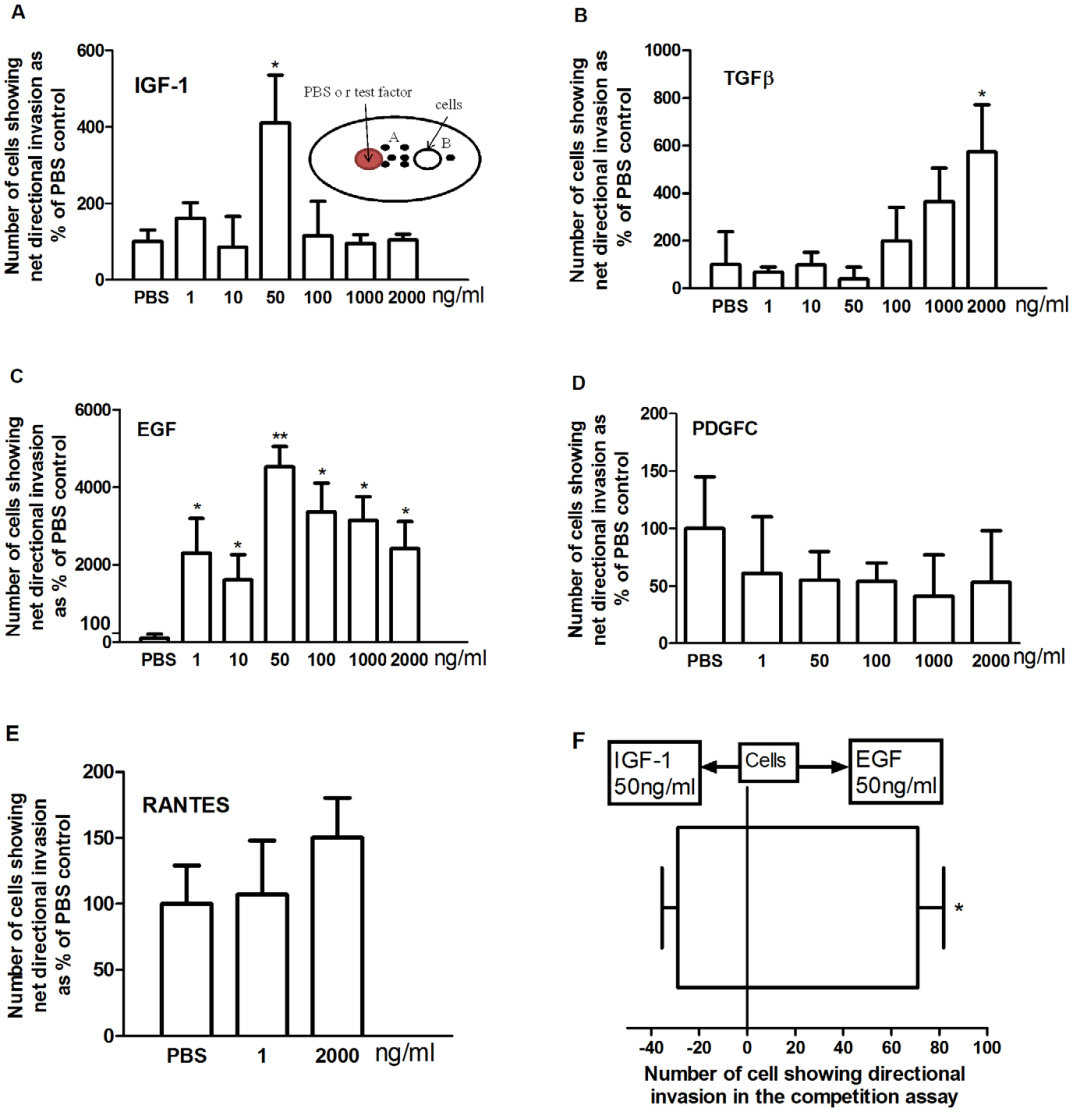
Effect of growth factors and chemokine on the directional invasive ability of pII cells. The net penetration of pII cells (A – B; see Fig 1) towards sources of IGF-1(A), TGFβ (B), EGF (C), PDGFC (D) and RANTES (E) at the concentrations indicated was determined in the absence of serum in the agarose but with addition of ITS as illustrated in the schematic diagram. PBS added in the left well served as control in each case with the cell count set at 100% and data for other conditions normalized to this. In another set of experiments (F), pII cells were placed in the middle one of three chambers formed in the agarose and allowed to move towards either IGF-1 or EGF (50 ng/ml) present in the two outer chambers. Histobars represent means ± SEM for 6–12 independent determinations. For panels A-C, asterisks denote significant difference over PBS control with p≤0.05 (*) and p≤0.001 (**). For Panel F movement towards EGF was significantly greater than towards IGF-1 with p<0.001 (*).

The effect of several growth factors known to affect breast cancer cells was examined. For these experiments, the agarose was prepared with ITS instead of 5% serum since some of these growth factors may themselves be serum constituents. We determined the net (directional) invasive movement of pII cells towards different concentrations of IGF-1, EGF, TGFβ, PDGFC and RANTES. pII cells were plated in one chamber and the growth factor/chemokine was added to another chamber 2.5 mm apart. The factor diffuses into the agarose layer forming a downward concentration gradient towards the cells [16]. Net directional invasion of cells towards the factor was calculated by subtracting the number of cells which moved towards the source of the stimulus from the number of cells which moved in the opposite direction (away from the chamber containing the stimulus). The data shown in Fig. 4-A illustrates a concentration – dependent bell-shaped curve observed for the net invasive movement of pII cells towards IGF-1. This resembles the classic dose response curve seen for other immune cell chemotaxis (neutrophils) when using this assay [16]–[18]. The optimal dose of IGF-1 inducing net invasion (3 fold increase over PBS) was 50 ng/ml. With TGFβ (Fig. 4-B), a significant increase in cell invasion was observed only at 2 µg/ml with lower concentrations showing no effect above the PBS control. EGF showed a very potent stimulatory effect even at 1 ng/ml (Fig. 4-C) reaching a peak at 50 ng/ml and declining thereafter in a bell shaped curve. The highest fold increase in the net invasion over PBS values was significantly greater than that observed with the other agents (300 fold increases for EGF vs. 3 fold increase for IGF-1 and TGFβ). PDGFC at similar doses did not induce pII cell directional invasion over the PBS control (Fig. 4-D) although these cells exhibit high expression of this growth factor [14]. The CC chemokine RANTES (Fig. 4-E) showed no enhancement in net invasion of pII cells tested at the same dose range as used for the growth factors. When pII cells (placed in a central chamber) were given a choice between two growth factors (IGF-1 vs. EGF; 50 ng/ml) present in the two outer chambers (competition assay, Fig. 4-F), these cells preferred to move in significantly higher numbers towards EGF, which is consistent with its greater potency in inducing pII cell invasion in comparison with the other growth factors tested. Neither IGF-1 nor EGF was able to induce invasion of the ER positive parental MCF-7 cells (data not shown).

### Effect of IGF-1, EGF, TGFβ, PDGFC and RANTES on the Random Invasion of pII Cells

**Figure 5:**
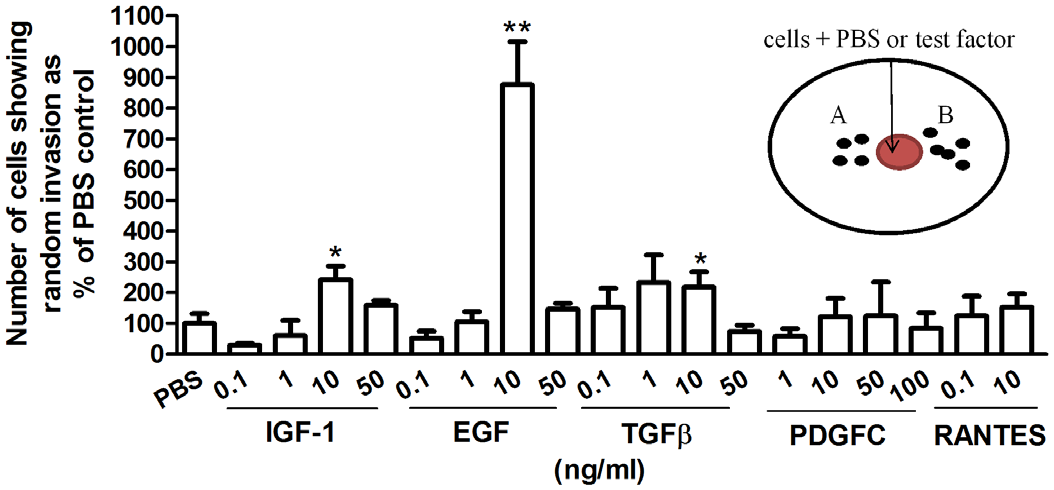
The effect of IGF-1, EGF, TGFβ, PDGFC and RANTES on the random (total) invasive movement of pII cells. A single chamber formed in 0.5% agarose containing ITS was loaded with 4×10^4^ cells in media containing either growth factor at the indicated concentration or an equivalent volume of PBS. After incubation for 24 h at 37°C cells that had moved into the agarose in either lateral direction were counted as described in Methods. Histobars represent means ± SEM of 4 independent determinations. Number of invading cells from the well given PBS was set at 100% and used as a normalizer. Asterisks indicate significant difference over PBS control with p≤0.05 (*) and p≤0.001 (**).

To exclude the possibility of different rates of diffusion between the growth factors/chemokine tested toward the cell-containing chamber which might result in different response rates, in these sets of experiments, the test factor was added directly to the chamber containing the cells and random (total) invasion was determined by counting cells that had penetrated into the agarose in either lateral direction out of the well. The dose of each agent used was lower than in the directional invasive experiments, in order to maintain some degree of approximate equivalence on the rationale that in this case cells were directly in contact with the agent instead of being exposed only to the diffused component. In terms of their relative effects, (at equimolar concentrations; 10 ng/ml) the order of potency of enhancement of the random invasion of pII cells was EGF >IGF-1> TGFβ. PDGFC and RANTES produced no effect above the PBS control (Fig. 5). There was no significant difference in cell growth between newly seeded control and growth factor treated cells within the 24 h period used for the invasion assays. Proliferation differences were usually seen at 2 days onwards and therefore not likely to contribute to differences observed in pII cell invasion.

### Effect of IGF-1, EGF, PDGFC and TGFβ on pII Cell Proliferation

**Figure 6:**
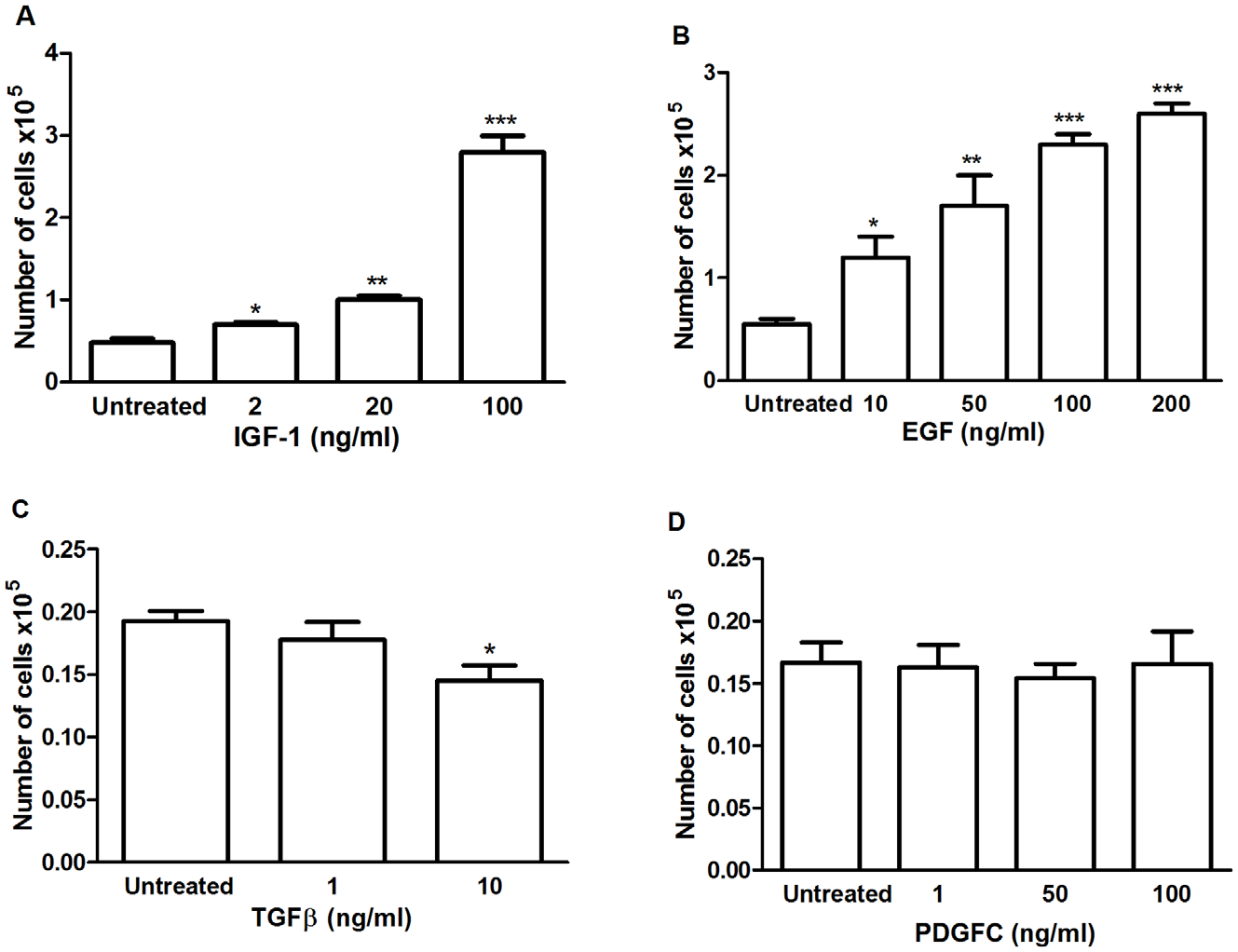
Effect of IGF-1, EGF, TGFβ and PDGFC on proliferation of pII cells. Approximately 10^4^ pII cells were seeded in microwell plates and allowed to attach in the presence of 5% serum. After 24 h the medium was changed to DMEM without serum with addition of IGF-1 (A), EGF (B), TGFβ (C) or PDGFC (D) at the indicated concentrations (vehicle was added to the control; untreated). Cells were harvested and counted after a further 4 days. Histobars represent means ± SEM of at least 3 independent determinations. Asterisks denote significant difference from untreated control with p = 0.01 (*), p = 0.0002 (**) and p = 0.0001(***).

The growth rate of pII cells was significantly increased in a concentration-dependent manner after treatment with IGF-1(Fig. 6-A) or EGF (Fig. 6-B) compared with the untreated cells, with a five-fold increase at 100 ng/ml (Fig. 6-A and B). On the other hand, TGFβ treatment resulted in significant inhibition at 10 ng/ml (Fig. 6-C). PDGFC showed no effect when used at similar dose range as for the other growth factors (Fig. 6-D).

### Effect of Erlotinib on pII Cell Invasion and Proliferation

**Figure 7:**
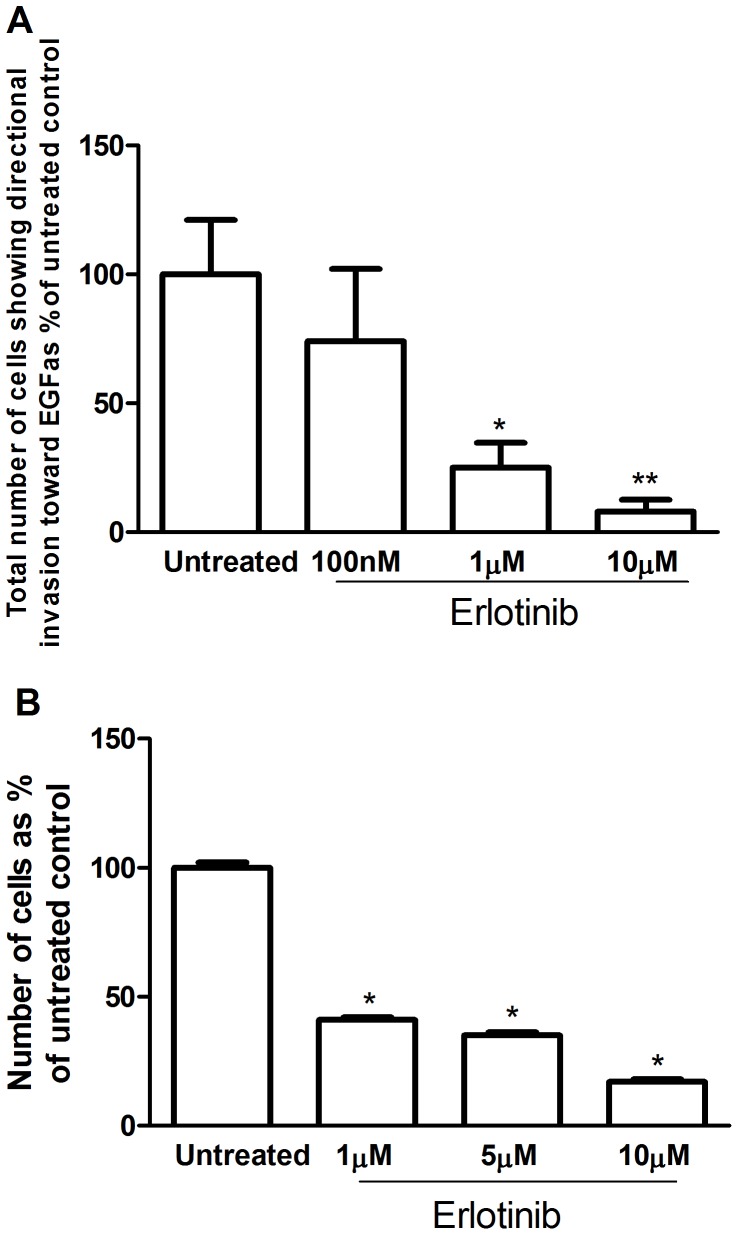
Effect of erlotinib on directional invasion and proliferation of pII cells. Panel A indicates the net directional invasive movement (A – B; as defined in Fig. 1) of pII cells through agarose towards a source of EGF (50 ng/ml) measured in the absence (untreated; UT) or presence of erlotinib added directly into the well containing cells as described in Methods. Data for drug treated conditions are expressed as a % of the untreated control (set as 100%). Each histobar is the mean ± SEM for 5 independent determinations. Asterisks denote significant difference from untreated control with p≤0.05 (*) and p<0.005 (**). Panel B shows the effect of erlotinib on cell proliferation. Approximately 10^4^ pII cells were seeded in microwell plates and allowed to grow over 4 days in the presence of various concentrations of erlotinib as indicated. Cells were harvested and growth was determined by the MTT assay. Histobars represent means ± SEM of at least 3 independent determinations. Asterisk denotes significant difference from untreated control, with p<0.001.

Erlotinib treatment significantly reduced the number of invading pII cells moving towards EGF (50 ng/ml) by 75% and 90% at 1 and 10 µM respectively (Fig. 7-A). It was also effective in the same concentration range in reducing cell proliferation (Fig. 7-B).

### Effect of IGF-1, EGF and TGFβ on Activation of Akt and ERK1/2

**Figure 8:**
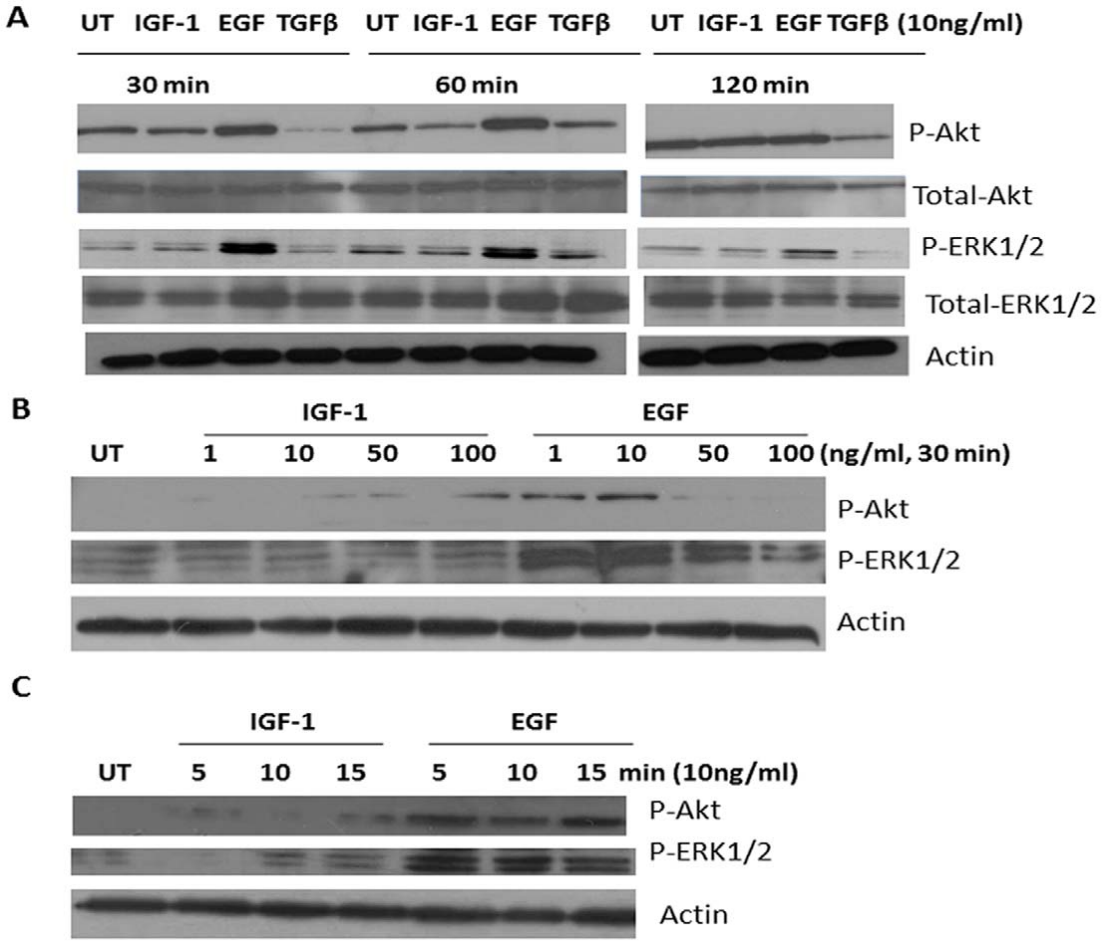
Effect of IGF-1, EGF and TGFβ on total and phosphorylated Akt and ERK1/2 in pII cells. Serum starved (24 h) cells were treated with the growth factors or vehicle (UT) for the times and at the concentrations indicated and subsequently harvested by scraping. Cell lysate protein (8 µg) was electrophoresed on 10% SDS polyacrylamide gel, blotted onto nitrocellulose membrane and probed with antisera to Akt, p-Akt, ERK 12, p-ERK1/2 and actin as described in Methods. Panel A shows levels of indicated proteins after 30, 60 and 120 min of exposure to the three growth factors or vehicle only. Panel B shows the effect of varying concentrations of IGF-1 and EGF treatment for 30 min on p-Akt and p-ERK 1/2 and Panel C the effect of shorter exposures to IGF-1 and EGF at a fixed concentration.

To determine the possible signaling events downstream of growth factor activation, we examined the degree of phosphorylation of Akt and ERK1/2, two important mediators that are known to be involved in pathways leading to proliferation and invasion as well as endocrine resistance [3]. As shown in Fig. 8-A, EGF treatment (10 ng/ml) significantly enhanced both Akt and ERK1/2 phosphorylation over a 30–120 min period while IGF-1 had no effect (Fig. 8-A). TGFβ treatment reduced Akt phosphorylation but had no effect on ERK1/2. There was no difference in total Akt or ERK1/2 levels in all of the conditions tested (Fig. 8-A). We subsequently tested various dose ranges and shorter stimulation times on the degree of Akt and ERK1/2 phosphorylation in response to IGF-1 and EGF treatment in pII cells. At 30 min (Fig. 8-B), a dose of IGF-1 at 100 ng/ml significantly enhanced the degree of Akt phosphorylation while EGF treatment enhanced Akt activity at a lower dose range (1–10 ng/ml, Fig. 8-B). ERK1/2 phosphorylation was not changed compared to the UT cells in response to IGF-1 stimulation even at higher doses (100 ng/ml), while EGF treatment enhanced ERK1/2 phosphorylation at 1–100 ng/ml dose range. We also measured Akt and ERK1/2 phosphorylation at shorter time points (5–15 min, Fig. 8-C) and found that both Akt and ERK1/2 phosphorylation was significantly enhanced in response to EGF compared to IGF-1 treatment with 10 ng/ml.

### Effect of Akt and ERK1/2 Signaling Pathways on EGF and IGF-1 Induced pII Cell Invasion

**Figure 9:**
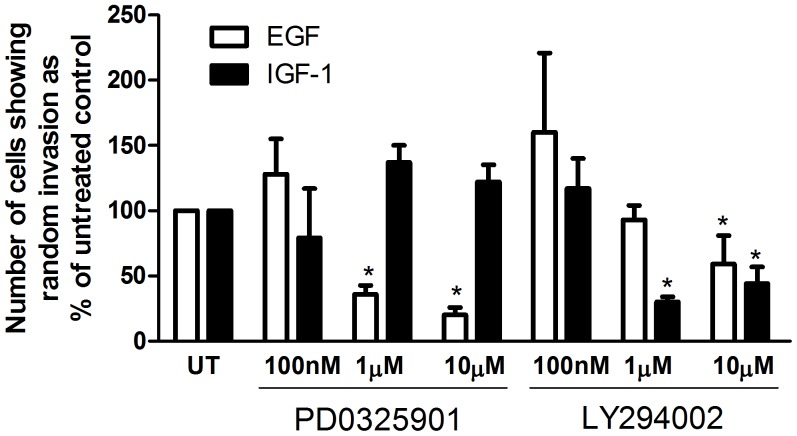
Effect of PI3K -Akt and ERK1/2 inhibitors on EGF and IGF-1 induced pII cell invasion. The random invasive movement (A + B; as defined in Fig 1) of pII cells in response to EGF (10 ng/ml) or IGF-1 (10 ng/ml) was measured in the absence (untreated; UT) or presence of the ERK1/2 inhibitor PD0325901 or the PI3K-Akt inhibitor LY294002 (at concentrations indicated) added directly to the cell medium. Data for drug treated conditions are expressed as a % of the untreated control (set as 100%) and are means ± SEM for 8 independent determinations. Asterisks denote significant difference from untreated control with p≤0.05.

To determine the significance of Akt and ERK1/2 phosphorylation in response to EGF and IGF-1 treatment, pII cells were pre-treated with the ERK1/2 inhibitor PD0325901 or the PI3K-Akt inhibitor LY294002 (100 nM-10 µM) and their random invasion in response to EGF or IGF-1 (10 ng/ml) was determined using the under agarose assay (Fig. 9). PD0325901 treatment did not affect the degree of invasion in response to IGF-1 while it significantly reduced the degree of pII cell invasion in response to EGF at 1 and 10 µM by 64 and 80% respectively (Fig. 9). On the other hand, LY294002 significantly reduced pII cell invasion in response to IGF-1 and EGF by 41–70%.

### Effect of IGF-1 and EGF Treatment on Matrix Metalloproteinase (MMP) Activity

**Figure 10:**
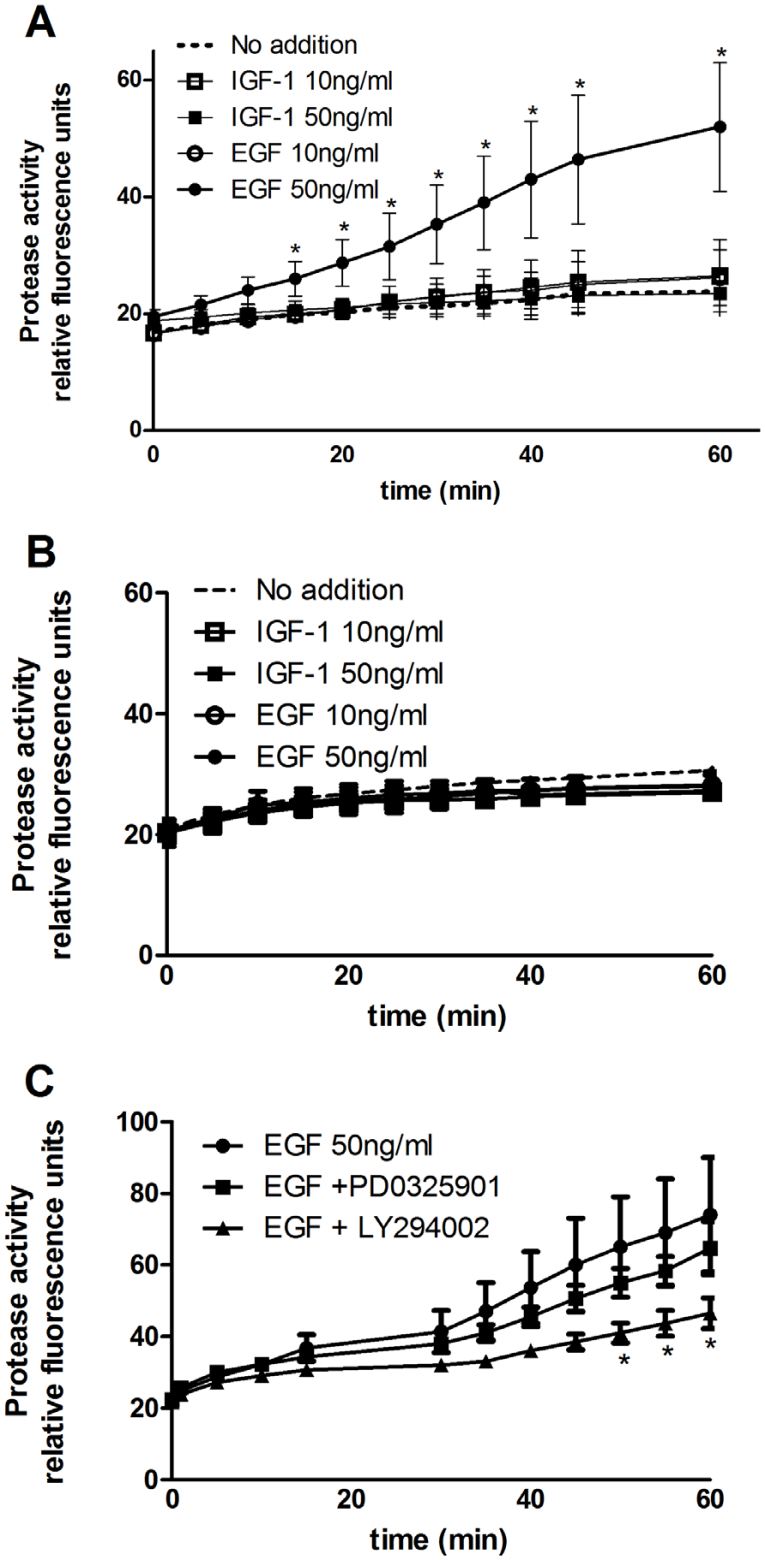
Effect of IGF-1, EGF and inhibitors on MMP activity. pII (A) or YS1.2 (B) cells were treated with IGF-1 or EGF (10 and 50 ng/ml) for 30 min and metalloproteinase activity was determined using a fluorogenic substrate as described in Methods. The fluorescence signal was monitored every 5 min for a total of 1 h using a microplate reader with excitation and emission at 490 and 525 nm respectively. Panel C shows the effect of pre-treatment of pII cells with 10 µM PI3K-Akt inhibitor LY294002 or ERK1/2 inhibitor PD0325901 on the EGF induced protease activation. Data are expressed as mean ± SEM of 3–6 independent determinations. In panel A asterisks denote significant difference between EGF at 50 ng/ml and no addition (p<0.05). In panel C asterisks denote significant difference between EGF alone and EGF+LY294002. (p≤0.05).

We studied the effect of IGF-1 and EGF treatment on the degree of extracellular (i.e. secreted) MMP activity which could explain how these growth factors can enhance the invasion of endocrine resistant breast cancer cells as indicated by the data in Figs. 4 and 5. As shown in Fig. 10-A, IGF-1 (10–50 ng/ml) had no significant effect on MMP activity as compared with the untreated controls, but interestingly EGF at 50 ng/ml significantly enhanced MMP activity over the controls (2.2 fold increase at 60 min). Interestingly, in the ER + cells YS 1.2 neither IGF-1 nor EGF had any effect on MMP activity (Fig. 10-B) compared to untreated control cells, which suggests a direct link between ER knockdown, increased MMP activity, and enhanced cell invasion.

In the presence of LY294002 there was a significant reduction in the degree of EGF-induced MMP activity in pII cells (38%) whereas PD0325901 produced a much smaller (14%) and non-significant effect (Fig. 10-C).

## Discussion

We studied the influence of various serum components on cell invasion and the modifying effects of erlotinib that is known to antagonize EGF action by inhibition of receptor tyrosine kinase activity. The HBL100 cells originally established from non-malignant breast cells were unable to penetrate into agarose. This was also the case for MCF-7 cells as has been shown before [14]. MDA-231and pII cells demonstrated a similar degree of random cell invasion that was dependent upon the presence of 5% FBS in the agarose. Interestingly, YS2.5 cells showed significantly enhanced degree of invasion compared to pII cells. Both lines were generated from MCF-7 transfection by ER directed shRNA constructs but the YS2.5 display a much greater ER knockdown effect (data not shown) suggesting that the invasive capacity of the cells may be influenced by the residual level of ER in previously ER expressing cells. A recent report by Ye *et al* [19] indicated that the degree of ER knockdown was related to increased invasion of MCF-7 cells into matrigel. They suggested a role for ER signaling in regulating E-cadherin expression and EMT through SNAIL2. The YS1.2 line, that was also derived by transfection with ER targeting shRNA but which did not exhibit reduced ER transcript levels, behaved similarly to the parental cells. This clearly demonstrates that it is the blockade of ER production that confers the ability to invade rather than transfection *per se*.

Although the combination of insulin, transferrin and selenium as a culture medium supplement was able to substitute the serum to some extent; it is apparent that other serum factors are needed for invasion. We examined the effect of four growth factors on the proliferation and migration of pII cells. Directional invasion was induced by addition of varying concentrations of these factors at a distance of 2.5 mm from the cell-containing chamber, or random invasion by addition of the growth factor directly into the well containing the cells. In both scenarios, the potency of EGF was greater than either IGF-1 or TGFβ.

IGF-1 has long been implicated in tumorigenesis as well as specifically in proliferation, survival and migration of tumor cells, with positive correlation to breast cancer progression [20], [21]. IGF-1 increases proliferation of ER positive and negative breast cancer cells [22], [23]. IGF-1 and EGF have previously been reported to enhance MCF-7 cell proliferation to a similar extent [23] and this equivalent potency was also observed in our pII cells (Fig. 6). In other studies, IGF-1 enhanced MCF-7 cell migration [24], [25] and invasion [26] with a bell-shaped dose-response curve in the same dose range used in our study, but with a different outcome. We observed that IGF-1 induced cell invasion in pII but not in MCF-7 cells (data not shown) in the under agarose assay.

TGFβ plays a complex role in carcinogenesis where it has a significant inhibitory effect on the growth of cancer cells at early stages but it induces EMT and enhances cell invasion ant metastasis at late stages [27], [28]. It is frequently over-expressed in breast cancer and its level of expression correlates with poor prognosis [29]–[31]. Its growth inhibitory effects have been noted in a wide variety of breast cancer cell lines [32]–[36] and such was also observed in our pII cells (Fig. 6-C). TGFβ has been reported to enhance the migration [10], [37], [38] and invasion [35], [38]–[41] of both ER-positive and negative breast cancer cells. In this study we found that TGFβ, while it did enhance pII cell invasion, it did so at a relatively high dose as compared with EGF and IGF-1, suggesting that at least its role in tumor invasion and metastasis is subsidiary to its effect as a growth inhibitory agent.

EGF and its receptor have been frequently associated with the progression of several forms of cancers including those of the breast [42], [43]. Although some studies have reported that EGF failed to induce proliferation of MDA-231 [44] or MCF-7 [45] another showed that it enhanced MCF-7 cell proliferation to a similar degree as IGF-1 [23] consistent with our own observation on pII cells. Several reports have confirmed the role of EGF in breast cancer cell migration [44], [46], [47] and invasion [47]. Our data suggest that EGF is the most potent enhancer of invasion among the growth factors tested. Our data also show that ER negative cells preferentially move towards a source of EGF when in competition with IGF-1 (Fig. 4-F). Furthermore, inhibition of EGFR activity by erlotinib profoundly inhibited pII cell invasion and proliferation (Fig. 7). These data support the potential benefits of selectively antagonizing EGF action particularly in early endocrine resistant breast cancer to prevent invasion and metastasis.

Some studies have suggested a role of the CC chemokine RANTES in inducing breast cancer cell migration [24], [48] and invasion [49]–[51], but we found no evidence to support this. RANTES failed to induce either directional or random invasion of pII cells at dosages similar to those used in the above mentioned studies.

A wide variety of growth factor induced downstream signaling molecules have been associated with breast cancer pathogenesis and shown to play vital roles in modulating processes such as cell growth, survival, EMT and invasion [6]–[11], [13], [52]–[55]. During this study we also compared the effect of imatinib, an agent that is thought to predominantly target the tyrosine kinase activity of the PDGF receptor, with suramin that has been used clinically as a broad spectrum growth factor antagonist. We found (data not shown) that imatinib was the most potent inhibitor of pII cell invasion and proliferation highlighting the importance of PDGF receptor as a potential therapeutic target to control breast cancer cell growth and metastasis [56]. Interestingly, Li *et al* [57] demonstrated a functional cooperation between EGFR and PDGFR during cell migration of murine fibroblasts whilst Abouantoun and MacDonald [58] suggested transactivation of EGFR by PDGFRB could be responsible for migration and invasion of medulloblastoma cells. They found that imatinib blocked PDGF-BB activation of PDGFRB, Akt and ERK and reduced the level of PDGFRB-phospho-EGFR heterodimers. In our previously reported study using microarray display [14] we did not observe any change in either PDGFB or PDGFRB mRNA in pII cells vs. MCF-7 but there was a significant elevation in PDGFA and PDGFD and a 50–200 fold increase in PDGFC. The latter was shown to interact with PGDFα and β receptors and has been described as a potent mitogen for mesenchymal cells [59]. As pII cells have acquired a mesenchymal-like phenotype [14] it may well be that imatinib is blocking the action of an over-expressed PDGFC. This drug is also clinically used to target the constitutive kinase activity of the BCR/ABL product, so it is uncertain which of these activities is responsible for our current observations. Therefore, we tested the effect of PDGFC but somewhat surprisingly, found that it had no effect on either proliferation or invasion. Moreover, PDGFC failed to show any synergistic effect on random invasion when added directly to cells together with sub-optimal concentrations of EGF, or in promotion of directional movement towards a source of EGF (data not shown). It remains to be determined if other PDGF isoforms such as A, B or D may play a role in enhancing breast cancer cell invasion.

The intracellular signalling pathways that can potentially transmit the effects of growth factors have been well described. In this study we looked at two central mediators (Akt and ERK1/2) and found that both were selectively activated by EGF, suggesting their involvement in promoting EGF-induced invasion. We also found that EGF significantly enhanced general MMP activity secreted by pII but not by the ER positive YS1.2 cells and that was mediated through the Akt (and in part through EKR1/2) pathway. We also showed that EGF-induced pII cell invasion was significantly blocked by the ERK inhibitor PD0325901 and the Akt-PI3K inhibitor LY294002 (Fig. 9) which is in agreement with our data generated from the MMP and western blotting experiments.

Although several reports demonstrated that IGF-1 can enhance MMP activity in both ER positive [25], [26] and ER negative [60] breast cancer cells, we did not observe any increase in general MMP activity with either pII or YS1.2 cells treated with IGF-1 at similar dose range used in these studies. Kim et al [61], [62] observed enhanced MMP-9 activity mediated through JAK3/ERK pathway in EGF treated SKBR3 breast cancer cells, whereas Park et al [63] showed enhanced MMP-1 activity through the involvement of ERK/MAPK signalling pathways. We observed a small (14%), but non-significant inhibition in general MMP activity following treatment of pII cells with the ERK inhibitor PD0325901 (Fig. 10-C). Our data is consistent with the findings of Lee et al [64] who showed that EGF enhances MMP-9 activity in the ER negative MDA-MB-231 cells mediated through the Akt-PI3K signalling pathway.

In summary, we have presented experimental evidence showing that both *de novo* and siRNA induced reduction of ER expression is directly associated with enhanced cell invasion. TGFβ plays a major role in inhibiting cell proliferation, whereas of the pro-proliferative factors IGF-1 and EGF, the latter plays a dominant role in cell invasion and by implication metastasis through activation of the PI3K and ERK pathways that we have previously suggested to be contributory mechanisms to endocrine resistance [3]. This is also underlined by the effectiveness of erlotinib indicating that this drug has potential therapeutic usefulness for preventing spread of particularly endocrine resistant breast cancer. The under agarose assay utilized in this study proved to be a simple and effective means of monitoring and quantifying cell invasion by permitting direct microscopic visualization as compared with methods that rely on indirect measurements of fluorescent signals emitted from post incubation of migrating cells with dyes such as calcein. It facilitates direct comparison of cell lines as well as simultaneous exposure of cells to more than one test factor.
